# Prevalence of diabetic retinopathy in Tehran province: a population-based study

**DOI:** 10.1186/1471-2415-9-12

**Published:** 2009-10-16

**Authors:** Mohammad Ali Javadi, Marzieh Katibeh, Nasrin Rafati, Mohammad Hossein Dehghan, Farid Zayeri, Mehdi Yaseri, Mojtaba Sehat, Hamid Ahmadieh

**Affiliations:** 1 Ophthalmic Research Center, Labbafinejad Medical Center, Shahid Beheshti University of Medical Sciences, Tehran, Iran; 2 Department of Biostatistics, Faculty of Paramedical sciences, Shahid Beheshti University of Medical Sciences, Tehran, Iran; 3 Department of Epidemiology and Biostatistics, School of Public Health, Tehran University of Medical Sciences, Tehran, Iran

## Abstract

**Background:**

To determine the prevalence and characteristics of diabetic retinopathy (DR) among Iranian patients with diabetes.

**Methods:**

Design: population-based cross-sectional study.

Participants: patients with diabetes aged 25 to 64 years in Tehran province, Iran. This survey was conducted from April to October 2007. The study sample was derived from the first national survey of risk factors for non-communicable disease. Diabetes mellitus was defined as a fasting plasma glucose of ≥ 7.0 mmol/l (126 mg/dl) or more, use of diabetic medications, or a physician's diagnosis of diabetes. All patients known to have diabetes underwent an eye examination by bio-microscope and indirect ophthalmoscope to check for any signs of DR through dilated pupils by + 78 lens. Participants were also interviewed and examined to determine their demographic characteristics, medical conditions and the regularity of their eye visits.

**Results:**

Among 7989 screened patients, 759 (9.5%) had diabetes. Of them, 639 patients (84.2%) underwent eye examination. Five patients (0.7%) with media opacity were excluded. Of 634 examined patients with diabetes, 240 had some degree of diabetic retinopathy, and the overall standardized prevalence of any retinopathy was 37.0% (95% CI: 33.2-40.8), including 27.3% (95% CI: 23.7-30.8) (n = 175) with non-proliferative and 9.6% (95% CI: 7.3-11.9) (n = 65) with proliferative diabetic retinopathy. Clinically significant macular edema and vision-threatening retinopathy were detected in 5.8% (95% CI: 4.0-7.7) (n = 38) and 14.0% (95% CI: 11.3-16.7) (n = 95) of patients, respectively. Only 143 patients (22.6%) with diabetes had a history of regular eye examination.

**Conclusion:**

This study demonstrated a high prevalence and poor control of DR in Tehran province. This suggests the need for adequate prevention and treatment in patients with diabetes.

## Background

Diabetes mellitus (DM) is one of the most common non-communicable diseases with an increasing incidence worldwide. Recent estimates indicate that there were 171 million people throughout the world living with diabetes in the year 2000, and this number is projected to increase to 366 million by 2030, with the most significant increase occurring in developing countries [1]. While most individuals affected with DM in developed countries are elderly, the majority of subjects in developing countries are younger (46-64 years of age), which intensifies the consequences of DM in these societies [2].

The main ocular complications of DM are cataracts and diabetic retinopathy (DR); the earliest clinical signs of DR present at different times depending on the diabetes type; they occur in nearly all subjects who have had type 1 diabetes (commonly early onset and due to immune-mediated factors) for 20 years [3] and in nearly 80 percent of those with type 2 disease (including individuals who are insulin resistant, obese and middle-aged and have relative insulin deficiency) with the same duration [4].

DR is increasingly becoming a major cause of blindness throughout the world; in addition, loss of productivity and quality of life for the patient with DR will lead to additional socioeconomic burdens on the community [5]. However, appropriate treatment can decrease the loss of vision caused by proliferative DR by up to 90% [6].

The type and duration of diabetes, age, gender, glycemic control, systemic hypertension, body mass index (BMI), smoking, serum lipids, and microalbuminuria are associated with the development and progression of DR [7-10].

According to a recent study in Tehran [11], the prevalence of DM is much greater than that in industrialized countries [5] (14% versus 2%) and about one-third of the patients with diabetes in Tehran [11] and half of those in Iran [12] are unaware of their illness. Furthermore, a study conducted in 2004 showed that DR is one of the most common causes of low vision and blindness in Tehran province [13]. A study in Isfahan, another large city in Iran, showed a high incidence of DR among patients with diabetes using a clinic information system (about 90 per 1000 person-years) [14]. Before the present study, no information was available on the prevalence of DR in a population based study in our country [15]; in addition, there is a lack of information about prevention and control of DR in Iran. Therefore, this study was conducted to determine the DR prevalence, characteristics and regularity of eye visits in a representative sample of patients with DM in Tehran province.

## Methods

This cross-sectional study was conducted from April to October 2007 in Tehran province. The study was approved by Iranian Center for Disease Control (CDC) regarding the methodology and ethical considerations. All investigations were performed according to the guidelines of the Declaration of Helsinki.

### Study sample

The study sample was derived from the first national survey of risk factors of non-communicable disease (SURFNCD) in Iran [12,16], which was performed in January and February 2005 using the guidelines of the stepwise approach to non-communicable disease risk factor surveillance of the world health organization [17]. In the SURFNCD, a multistage probability cluster sampling scheme was used for random selection of adults throughout the country. Using a stratified random cluster sampling in Tehran province, proportional to the size of each district, 9993 citizens were selected from 500 clusters. Cluster sampling was based on block sampling in urban areas and family charts in rural areas. In each cluster, after random selection of the first house as the index, houses on the right side of the index were selected to fulfill a total of 20 individuals in each cluster. Five different age groups (15-24, 25-34, 35-44, 45-55 and 55-64 years old) with four individuals (2 men and 2 women) in each group were defined in all clusters. All participants aged 25 to 64 years (7989 persons) were invited to the national diabetes screening program. Participants who had reported a history of diabetes diagnosed by a physician or health care professional were classified as known diabetics. Participants were asked to go to a specified laboratory for collection of blood samples, following a 12-hour fast to identify the undiagnosed patients. Ultimately, 759 patients with diabetes were defined in Tehran province, of whom 502 (66.1%) had reported a history of known diabetes.

### Data collection

Invitation for the current study was sent to all registered patients with diabetes. Two follow-up notes were sent to those who failed to respond to the initial invitations; in addition, all related expenses, including medical evaluation, treatment and transportation were covered by the research group. Those who did not respond after the third invitation were considered non-responders.

In the next step, all participants were referred to an ophthalmic clinic (Negah Eye Clinic), under supervision of the Ophthalmic Research Center. Participants were interviewed and examined to determine their demographic characteristics and medical conditions in addition to their medications and diet. Required data were collected, including: sex, age, disease duration, type of diabetes, dyslipidemia (based on laboratory findings or use of any lipid-lowering drugs), and history of diagnosed nephropathy. In addition, two close-ended questions assessed the regularity of ophthalmic assessment by an ophthalmologist and the source of information for patients with regular ophthalmic visits. The term "regular visits" was defined as the annual ophthalmic examination in accordance with the definition of American diabetes association.

All patients were examined by a single retina sub-specialist who had a five-year work experience in this field and a good inter-observer agreement with an expert retina sub-specialist (HA) for diagnosis and grading of DR (both Kappa values were greater than 0.9). A supervisor team trained the examiner at the beginning of the study and evaluated her during the project.

Complete eye examinations were performed for the patients. Uncorrected and best corrected visual acuities were determined using Snellen charts and a Topcon autorefractometer (KR 8000, Topcon, and Tokyo, Japan). The ophthalmologic evaluation included bio-microscope examination of the anterior segment, lens opacity, intraocular pressure measurement using Goldmann applanation tonometry (Haag-Streit, Bern, Switzerland) and dilated fundoscopy using a +78-diopter non-contact fundus viewing lens (Nikon). Finally with examination by indirect ophthalmoscopy, a full retinal and macular examination was completed. DR was graded according to the scale proposed by the American academy of ophthalmology [18] based on the more severely affected eye. Vision-threatening retinopathy was defined as the presence of severe NPDR, PDF or CSME [19].

Patients with media opacity significant enough to preclude the retinopathy evaluation were excluded from the analysis. All instruments were regularly calibrated at the beginning and during the study.

### Definitions

Diabetes mellitus was defined as use of diabetic medication or a physician's diagnosis, or, in those without known diabetes, it was defined as fasting plasma glucose ≥ 7.0 mmol/l (126 mg/dl) in accordance with the current WHO diagnostic criteria for diabetes [20]. However, given that oral glucose tolerance testing (2-h plasma glucose ≥ 11.1 mmol/l (200 mg/dl)) was not performed in the original survey, we did not include this item in the case definition. Glycosylated hemoglobin (HbA1c) level was measured using the Bayer DCA 2000+ analyzer, with values less than 7% considered to be indicators of good glycemic control.

Blood pressure was measured in the seated position using standard mercury sphygmomanometers, and hypertension was defined as a systolic blood pressure of 140 mmHg or more and/or a diastolic blood pressure of 90 mmHg or more; or ongoing treatment with antihypertensive drugs [2]. Hyperlipidemia was defined as total cholesterol of 6.2 mmol/l or more or the use of lipid-lowering drugs [21].

Visual impairment was classified based on best corrected visual acuity (BCVA). Low vision was defined as BCVA less than 20/60, but equal to or better than 20/400 in the better eye, and blindness was defined as visual acuity less than 20/400 in the better eye based on WHO criteria [22].

### Statistical analysis

For descriptive purposes, quantitative variables were presented as mean ± SD, and qualitative data were reported in terms of rates and proportions. In addition, age-sex adjusted prevalence rates and their confidence intervals (CI) 95% were reported. For analytic purposes, convenient parametric and non-parametric analyses such as Chi-square, Fisher's exact and Mann-Whitney tests were utilized. To evaluate the simultaneous effect of different risk factors or risk indicators including age, gender, duration of diabetes, hypertension, HbA1C, nephropathy, hyperlipidemia and method of diabetes control on the presence of DR (the response variable), a multiple logistic regression model was used. P-values less than 0.05 were considered statistically significant. Statistical analysis was performed using STATA version 8 software.

## Results

Among 7989 screened individuals, 759 (9.5%) had diabetes. Of them, 639 eligible patients (a response rate of 84.2%) participated in this study. Five patients (0.7%) were excluded due to severe corneal or lens opacity precluding fundus examination. The mean ± SD age of the 634 remaining patients was 58.16 ± 11.98 years.

Ophthalmic examination revealed that 240 subjects had some degree of DR (age standardized prevalence rate of 37.0%, 95% CI: 33.2-40.8), including 175 patients with non-proliferative (NPDR) and 65 patients with proliferative diabetic retinopathy (PDR). Clinically significant macular edema (CSME) was detected in 38 patients. Among patients with CSME, 20 patients had NPDR (11.4% of the NPDR patients), and 18 had PDR (27.7% of PDR patients). See Table 1 for more detailed information about the crude and age-standardized prevalence rates of different grades of DR and CSME in the patients with diabetes in this study. The presented P-values in Table 1 show that there is a significant difference between male and female patients in age-standardized prevalence rates of different grades of DR and CSME.

**Table 1:** Prevalence and severity of diabetic retinopathy and macular edema by gender

	**Total persons with diabetes** **(N = 634)**			**Men with diabetes** **(N = 287)**			**Women with diabetes** **(N = 347)**			**P^¶^**
		
	**n**	**Crude†**	Standardized*	**n**	**Crude†**	Standardized*	**n**	**Crude†**	Standardized*	
Any retinopathy	240	37.9 (34.1-41.6)	37.0 (33.2-40.8)	124	43.2 (37.4-49)	43.9 (38.1-49.6)	116	33.4 (28.4-38.4)	33.1 (28.1-38.0)	0.005
CSME^§^	38	6.0 (4.1-7.8)	5.8 (4.0-7.7)	20	6.9 (3.9-9.8)	6.9 (4.0-9.8)	18	5.2 (2.8-7.5)	5.3 (2.9-7.6)	<0.001
VTR^§^	95	15 (12.2-17.8)	14.0 (11.3-16.7)	58	20.2 (15.5-24.9)	21.9 (17.1-26.7)	37	10.7 (7.4-13.9)	9.5 (6.4-12.6)	<0.001

**Retinopathy Grades**										
NPDR	175	27.6 (24.1-31.1)	27.3 (23.7-30.8)	85	29.6 (24.3-34.9)	29.3 (24.0-34.6)	90	25.9 (21.3-30.6)	26.2 (21.6-30.9)	<0.001
Mild NPDR	114	17.8 (14.9-20.8)	17.9 (14.9-20.9)	53	18.2 (13.8-22.7)	17.7 (13.3-22.1)	61	17.5 (13.5-21.5)	17.9 (13.9-22.0)	<0.001
Moderate NPDR	42	6.6 (4.6-8.5)	6.6 (4.7-8.6)	20	6.9 (3.9-9.8)	6.4 (3.6-9.2)	22	6.3 (3.8-8.9)	6.7 (4.1-9.4)	0.024
Severe NPDR	19	3 (1.7-4.3)	2.6 (1.4-3.9)	12	4.1 (1.8-6.4)	4.8 (2.3-7.2)	7	2 (0.5-3.5)	1.4 (0.1-2.6)	<0.001
PDR	65	10.3 (7.9-12.6)	9.6 (7.3-11.9)	39	13.6 (9.6-17.6)	14.5 (10.4-18.6)	26	7.5 (4.7-10.3)	6.8 (4.1-9.5)	<0.001

†Data presented in Crude and Standardized columns are percentages and confidence intervals 95%.

* Age- standardized to the 2006 Tehran population census.

^¶ ^P- Value for difference in prevalence and severity of retinopathy by gender, based on chi-square test.

§CSME: Clinically significant macular edema, VTR: Vision threatening retinopathy

Table 2 shows the frequency distribution and the prevalence rate of DR of all grades by different characteristics of the patients. Univariate statistical tests revealed that there was a significant relationship between the presence of DR and patients' age, sex, duration of diabetes, presence of hypertension and nephropathy, and method of diabetes control. The P-values presented in the last column of Table 2 show the results of statistical tests evaluating the differences between characteristics of male and female patients with any grade of DR. The only significant P-value in this column tells us that female patients with DR had a higher rate of hypertension than males with DR [69 out of 116 women (59.9%) with DR vs. 38 out of 124 men (30.6%) with DR, P < 0.001].

**Table 2:** Participant characteristics by presentation of diabetic retinopathy and gender

**Characteristic**	**Total** **(N = 634)**	**No DR** **(n = 394)**	**Any DR** **(n = 240)**	P*	**Men with DR** **(n = 116)**	**Women with DR** **(n = 124)**	**P^¶^**
**Age (years)**	59.29 (11.98)	60.15 (12.50)	55.89 (10.96)	0.017	57.73 (10.56)	58.06 (11.42)	0.814
**Sex (%)**							
Male	287 (45.3)	163 (56.8)	124 (43.2)	0.012	-	-	-
Female	347 (54.7)	231 (66.6)	116 (33.4)				
**Diabetes type**							
I	16 (2.5)	7 (43.8)	9 (56.3)	0.12	3 (42.9)^§^	6 (66.7)^§^	0.321
II	618 (97.5)	387 (62.6)	231 (37.4)		121 (43.2)	110 (32.5)	
**Duration (year)**							
<5	215 (33.9)	167 (77.7)	48 (22.3)	<0.001	27 (29.7)	21 (16.9)	0.981
5-10	214 (33.8)	141 (65.9)	73 (34.1)		32 (34.0)	41 (34.2)	
11-15	100 (15.8)	54 (0.54)	46 (0.46)		28 (59.6)	18 (34.0)	
15-20	47 (7.4)	12 (25.5)	35 (74.5)		19 (86.4)	16 (64.0)	
>20	58 (9.1)	20 (34.5)	38 (65.5)		18 (54.5)	20 (80.0)	
**Hypertension**							
Absent	383 (60.4)	250 (65.3)	133(34.7)	0.045	86 (42.6)	47 (26.0)	<0.001
Present	251 (39.6)	144(57.4)	107 (42.6)		38 (44.7)	69 (41.6)	
**Nephropathy**							
Absent	599 (94.5)	380 (63.4)	219 (36.6)	0.005	112 (42.1)	107 (32.1)	0.599
Present	35 (5.5)	14 (40.0)	21 (60.0)		12 (57.1)	9 (64.3)	
**Hyperlipidemia**							
Absent	360 (56.8)	232 (64.4)	128(35.6)	0.17	73 (39.9)	55 (31.1)	0.075
Present	278 (43.2)	162(59.1)	112 (40.9)		51 (49.0)	61 (35.9)	
**HgbA1C**							
Control	396 (62.5)	252 (63.6)	144 (36.4)	0.318	68 (40.7)	76 (33.2)	0.092
Uncontrol	238 (37.5)	142 (59.7)	96 (40.3)		56 (46.7)	40 (33.9)	
**Diabetes control**							
Diet +Exercise	56 (8.8)	55(98.2)	1(1.8)	<0.001	0 (0.0)	1 (2.6)	0.319
Oral medication	513 (81.0)	309 (60.2)	204 (39.8)		103 (43.8)	101 (36.3)	
Insulin injection	65 (10.2)	30(46.2)	35 (53.8)		21 (61.8)	14 (45.2)	

Data presented are means ± standard deviation for age or number (%) for other characteristics.

* P- Value for difference in characteristics by Diabetes Retinopathy (DR) status, based on chi-square test, Fisher's Exact, Mann-Whitney or t-test, as appropriate.

^¶^P- Value for difference in characteristics by gender in patients with DR based on chi-square, Fisher's Exact, Mann-Whitney or t-tests, as appropriate.

^§^Percentages in two columns showing men/women with DR concern the percentage of each characteristic in comparison with men/women without DR which are omitted to make the table less crowded

The prevalence of low vision and blindness among 634 participants were 6.5% (95% CI: 4.7-8.7) and 1.6% (95% CI: 0.8-2.9), respectively. The prevalence of any type of visual impairment (BCVA < 20/60) in patients with PDR was remarkably higher than that in patients without PDR (18.5% vs. 7.0%, P = 0.002, OR = 2.09, 95% CI: 1.02-4.26).

Among the diabetic patients studied (634 cases), 233 patients (36.8%) had a history of eye examination. Of them, only 143 patients (22.6%) had a history of regular eye examination, while 90 patients (14.2%) reported non-regular ophthalmologist visits. The other 401 patients (63.2%) said that they would have an eye examination after the occurrence of an ocular symptom. The prevalence of DR in the above mentioned groups was 18.9%, 30.0% and 46.4% respectively (P < 0.001). Among patients with regular eye examination (143 patients), 61.1% had been informed to do so by physicians, 17.4% by mass media and 21.5% by other patients with diabetes.

In the final step, to assess the simultaneous effect of different risk factors or risk indicators on the presence of any DR, a logistic regression model was utilized (Table 3). The obtained results revealed that males, patients with a longer history of diabetes, patients using insulin or oral medication for diabetes control, and patients with hypertension or nephropathy had a statistically significant increase in risk of any grade of DR as compared to other subjects.

**Table 3:** Risk factors for diabetic retinopathy based on logistic regression results

	OR*	**95% CI**	**P**
**Age (Per 10 yrs)**	0.95	0.93-0.96	<0.001
			
**Sex (Male gender)**	1.53	1.05-2.23	0.025
			
**Duration (year)**			
>20	9.75	4.44-21.37	<0.001
15-20	6.99	3.45-14.1	<0.001
11-15	2.60	1.49-4.53	0.001
5-10	1.84	1.18-3.04	0.008
<5		Reference	
			
**Hypertension**	1.55	1.04-2.29	0.028
			
**Nephropathy**	2.05	1.08-3.83	0.04
			
**Hyperlipidemia**	1.03	0.71-1.50	0.872
			
**Uncontrolled HgbA1C**	1.05	0.71-1.49	0.793
			
**Method of diabetes control**			
**Insulin use**	32.71	4.11-259.85	0.001
**Oral medication**	28.82	3.8-213.69	0.001
**Diet+ exercise**		Reference	

* Multivariate odds ratios which are adjusted for age, gender, duration of diabetes, hypertension, HbA1C, nephropathy, hyperlipidemia and method of diabetes control.

## Discussion

The present study showed that the prevalence of DR in a representative sample of patients with diabetes in Tehran in 2007 was 37%. This prevalence is comparable to findings obtained from non-Asian populations [23,24] and two other major Asian population-based studies in Taiwan (35%) [25] and Singapore (35%) [26]. In addition, a clinic-based study in Oman reported a comparable prevalence (42%) [27]. Most of the Asian studies indicated a much lower prevalence of DR, some of these studies, such as the Chennai urban rural epidemiology study (CURES) [28], were population based and used retinal photography which indicated 17.6% and 5.1% DR in known and newly diagnosed diabetics respectively. Another population based study from the United Arab Emirates reported 19% DR prevalence [29]. Some studies used clinical examinations such as reports from Pakistan and India, which demonstrated 17.5% and 26.2% DR prevalence respectively [30,31]. These differences may be due to variations in setting, sample size, limitation in compensation of confounders and the diagnostic method (imaging vs. clinical) [32]. However it seems the DR prevalence is lower in some ethnicities in Asia than it is in Caucasians [33].

In the current study, men, as compared to women, had significantly higher prevalence with greater severity of diabetic retinopathy in both univariate and multivariate analysis. A similar male preponderance has been reported in some studies [[28,29,34] and [35]]. In contrast, other studies have not shown a consistent pattern of gender variation in DR prevalence [[3,23,26] and [36]] or incidence [37]. In the Singapore Malay Eye Study, a higher prevalence of more severe DR was observed in women; however, this difference was lost after adjustment for metabolic and socioeconomic risk factors [26]. More studies are needed to examine the causes of this inconsistency of DR prevalence in gender differences in different populations.

The prevalence of macular edema in our study (5.8%) is comparable to the findings obtained from previous reports [26,36]. In addition, the present study confirmed the correlations found in other studies between risk factors such as longer duration of diabetes, systemic hypertension and nephropathy and the presence of DR [23-30,34-39].

Recently, a comprehensive systematic review revealed that tight glycemic control (HbA1c in normal range) reduces the incidence and progression of DR [38]. In the present study, the quality of glycemic control did not show any significant association with DR. This unusual finding may be a manifestation of a better blood sugar control after the diagnosis of diabetes through the national survey three years ago.

Visual impairment and blindness were observed in 8.1% of our patients; these rates were much higher than the percentage seen in the adult population in Iran. A recent comprehensive population-based study conducted in a similar setting, age group and using a similar definition for visual impairment in a normal population in Tehran province showed a 1.67% prevalence of visual impairment [40]. Furthermore there is a 5.7% estimate for visual impairment in the eastern Mediterranean region in the population over 50 years of age [22]. This finding shows poor screening and management in patients with diabetes in our population, given that studies have shown that loss of vision due to diabetes is uncommon in a population that is carefully screened for diabetes mellitus and provided with regular eye screening [6,41]. We found a high preponderance for visual impairment in patients with PDR which is in line with previous findings [42].

Routine and repetitive clinical retinal examination is essential for the fundamental ophthalmic care of patients with diabetes [43]. Retinopathy screening should be performed within three to five years after the onset of type 1 diabetes and shortly after the diagnosis of type 2, with annual follow-up examinations in both types of diabetes [44]. In contrast with developed countries [6,41], most of the patients with diabetes (81.1%) in this study had no regular follow up program for management of DR and the prevalence of DR was found to be higher in these patients. In addition, it should be mentioned that samples of this study were enrolled from a previous survey which was performed 3 years prior to the current study. During that study 35% of the patients who were unaware of their diabetes at the time were informed about their diseases and might be told to have regular eye examination. So the real mismanagement of retinopathy is probably even higher in society than the results of this study show.

In summary, the present study is the first report of DR from Iran via a population-based study. By extrapolating the adjusted prevalence rates, we can estimate that 237000 people out of about 640000 adults with diabetes in Tehran province have some degree of DR and 14 in 100 adults with diabetes have vision-threatening DR.

There are some limitations in this study that might be important for interpretation of results. First of all, the baseline data of non responders (16%) was unavailable; this could be a potential source of bias. In addition, the prevalence of nephropathy in our study might be lower than the true prevalence, as we only determined nephropathy by a history of a previous diagnosis. It should be mentioned that our definition of hypertension differs from that of the American physician association from 2007, which defines appropriate blood pressure as less than 130/80 mmHg in diabetic patients; however, some of the major population-based surveys [[25,26] and [45]] defined appropriate blood pressure in patients with diabetes as 140/90 mmHg. Only 2.5% of diabetes cases in this study had type I diabetes mellitus, this limited number did not allow us to compare type I and type II diabetes. Finally, we used clinical examination for diagnosis and grading of DR. While clinical exam is inexpensive and widely available, it is not very sensitive when compared with stereoscopic fundus photography and could limit a direct comparison with other similar recent studies.

## Conclusion

The prevalence of DR in Tehran province was 37%. Significant risk factors for DR were: male sex, long duration of diabetes, oral medication or insulin use, presence of systemic hypertension and nephropathy. The results of the present study show that eye care for many of patients with diabetes is insufficient. Most of the patients in this study had no regular ophthalmic assessments and the prevalence of DR was found to be higher in these patients. In this context, regular screening in patients with diabetes for early detection of proliferative retinopathy and increasing public awareness are highly recommended.

## Competing interests

The authors declare that they have no competing interests.

## Authors' contributions

MAJ suggested the initial concept and provided administrative and technical support for conducting the study. MK and MS contributed to the study design and did critical revision of the manuscript for important intellectual content. NR contributed to the study design by preparing the final report and drafting the manuscript. MHD participated in the eye examinations. FZ and MY performed the statistical analysis and interpretation of data. HA provided the technical and material support for the study and supervised the data collection. All authors read and approved the final manuscript.

## Pre-publication history

The pre-publication history for this paper can be accessed here:


